# The Influence of Wood-Derived Compounds on the Quality of Alcoholic Beverages

**DOI:** 10.3390/molecules31091408

**Published:** 2026-04-24

**Authors:** Paweł Sroka, Tomasz Tarko

**Affiliations:** 1 Department of Fermentation Technology and Microbiology, University of Agriculture in Krakow, ul. Balicka 122, 30-149 Krakow, Poland; 2 Centre for Innovation and Research on Prohealthy and Safe Food, University of Agriculture in Krakow, ul. Balicka 104, 30-149 Krakow, Poland

**Keywords:** alcoholic beverages, barrels, maturation, oak wood, pyrolysis, toasting, phenolic compounds

## Abstract

Wood is a material frequently used in the production of alcoholic beverages. Oak barrels have been used to store wines, beers, and spirits for generations. Nowadays, beverages are increasingly matured in the presence of staves and chips from various wood species. The aim of this article was to describe the impact of different wood species and their thermal processing conditions on the quality of alcoholic beverages. The article describes the chemical composition of wood and the compounds formed during toasting at various temperatures. It also lists the volatile compounds extracted from wood for alcoholic beverages, along with their sensory thresholds and their impact on olfactory sensations. Attention was drawn to potentially harmful substances formed during wood toasting, including aromatic hydrocarbons and polycyclic aromatic hydrocarbons. The amounts of compounds extracted from wines, beers, and spirits from different wood species and toasted under different conditions were compared. *Quercus* barrels contribute to higher lactone concentrations in beverages, which have a coconut aroma. Cherry, acacia, and ash wood increase the concentration of volatile phenols in beverages. The use of staves and chips shortens the maturation time and facilitates the design of beverages with specific sensory characteristics.

## 1. Introduction

Barrels have been used since antiquity as containers for various raw materials, primarily for beverages. They were used to store and transport wines, beers, and distilled spirits. Even then, it was observed that wines and other drinks stored in barrels exhibited improved sensory attributes, a distinctive color, greater durability, and more straightforward clarification [1,2,3,4]. For this reason, barrels have been considered “active packaging.” The sensory properties of beverages associated with wood depend on many factors, including the wood-beverage exchange balance related to the wood’s sorption mechanism, the barrel production process, and the composition of the maturing beverage. However, the most crucial factor is the wood’s composition [2,5,6,7]. Other factors are also important, such as the duration of contact with the beverage, the contact surface, temperature, ethanol content, and pH. Critical parameters include the degree of wood toasting, which depends on exposure temperature and duration [8]. Historically, barrels were made from white oak, red oak, chestnut oak, red or sweet gum, sugar maple, yellow or sweet birch, white ash, Douglas fir, beech, black cherry, sycamore, redwood, spruce, bald cypress, elm, and basswood in the U.S. In Europe, oak, chestnut, fir, spruce, pine, larch, ash, mulberry, and other exotic woods from Africa, Australia, and South America [3,9]. Currently, due to its properties: tightness, thermoplasticity, durability, grain, oak wood is mainly used, especially the varieties *Quercus petraea* (Sessile oak), *Quercus robur* (Pedunculate oak), and *Quercus alba* (American oak), but also other species of trees, e.g., chestnut, cherry, acacia [3,5,7,10,11,12].

## 2. Objectives

The aim of this article was to describe the impact of different wood species and their thermal processing conditions on the quality of alcoholic beverages. The article describes the chemical composition of wood and the compounds formed during toasting at different temperatures. Volatile compounds extracted from wood for alcoholic beverages were summarized, along with their sensory thresholds and effects on olfactory perception. Attention was also paid to potentially harmful substances produced during wood toasting. The amounts of compounds extracted from wines, beers, and spirits made from different types of wood and toasted under different conditions were compared.

## 3. Methods

This review assessed the literature using the Scopus and Web of Science databases for manuscripts published through December 2015. Primary literature was considered, along with review articles and textbooks relevant to the topic. The cited articles were published in English.

## 4. The Chemical Composition of the Wood Used to Make Barrels

The wood used to produce oak barrels should have appropriate mechanical properties, ensuring strength, durability, and tightness, while also maintaining permeability to gases (mainly oxygen), ethanol, and water vapor [13,14]. The chemical composition of wood is variable and depends on the species of tree from which it was obtained, the region of cultivation, silvicultural treatment, climate, seasoning conditions, and wood processing [14]. On the other hand, this material contains numerous chemical compounds that can be extracted during beverage production and storage [15]. The mechanical properties of wood result from the presence of tensile-resistant cellulose fibers, hemicelluloses, and lignins, which provide adequate stiffness. These compounds are poorly soluble in water and alcoholic solutions. The two complex carbohydrates mentioned above, cellulose and hemicellulose, constitute 65 to 75% of wood. The third biopolymer, lignin, constitutes 20 to 28% of wood mass and consists of p-coumaryl alcohol units and their derivatives: coniferyl alcohol and sinapic alcohol [16,17].

Wood fractions soluble in water and alcohol solutions constitute from 2 to 5% and consist mainly of polyphenols (tannins, lignans), fats, organic acids, waxes, resins, terpenes, terpenoids, and minerals [18,19,20]. The last group includes salts of elements such as calcium, potassium, and magnesium, whose amounts do not exceed 1%. Increasing the ethanol concentration during wood extraction significantly increases the amount of extracted substances [19].

During barrel production, oak staves are heated to high temperatures to soften them and facilitate mechanical processing. Numerous physical and chemical reactions occur under these conditions. At temperatures up to 150 °C, water and low-molecular-weight volatile compounds evaporate primarily from the wood. In anaerobic conditions, hemicelluloses decompose at temperatures between 220 and 315 °C, cellulose decomposes at temperatures between 315 and 400 °C, and lignin decomposes at temperatures between 160 and 900 °C [20,21].

Hemicelluloses are a group of polysaccharides and their derivatives (e.g., uronic acids) that form branched molecules linked by β-glycosidic bonds. In addition to glucose, they contain other simple sugar residues such as xylose, mannose, arabinose, and galactose [22]. The chemical composition of hemicelluloses depends mainly on the hardness of the wood. Softwood contains galactoglucomannan (5–8%) of wood; arabinoglucuronoxylan (7–10%) of wood; and arabinogalactan (5–35%) of wood, while hardwood is dominated by glucuronoxylan (15–30%) of timber and glucomannan (2–5%) of wood [15]. Due to their lower degree of polymerization and irregular structure, hemicelluloses are less chemically resistant and more susceptible to degradation than other biopolymers present in wood. These compounds occur in amounts ranging from 25% in softwoods to 35% in hardwoods [13]. Chemical processing of hemicelluloses yields furan compounds and organic acids. Glycosidic bonds undergo hydrolysis, releasing monosaccharides and uronic acids. Under these conditions, monosaccharides undergo caramelization and Maillard reactions. Glucuronic acid and 4-O-methyl-glucuronic acid undergo decarboxylation and dehydration. Aldehydes (acetaldehyde, glycolaldehyde, methyl glyoxal), furan compounds, and low-molecular-weight organic acids such as acetic, formic, and glycolic acids are produced [23]. Carbonyl compounds can react with each other to form dimers, trimers, and other oligomers that form hydrates with water and compounds with other substances [24]. Increasing the pyrolysis temperature changes the qualitative and quantitative composition of the resulting compounds. At temperatures above 275 °C, cellulose also degrades and depolymerizes, while intermediate products dehydrate, producing furan compounds, aldehydes, and low-molecular-weight organic acids (acetic acid) [20].

Cellulose occurs in wood in an amount of 40 to 50% of the dry substance [13]. It is a biopolymer and is relatively thermally stable [25]. Hemicelluloses and lignin constitute the amorphous phase of wood, while cellulose constitutes the crystalline phase. The degree of cellulose crystallinity increases as wood is heated [26]. At temperatures above 160 °C, thermal degradation of wood biopolymers occurs, reducing their average molecular weight [25]. Cellulose pyrolysis mainly produces furans, pyrans, and linear small molecules (Table 1). The foremost pyrolysis products include levoglucosan, glycolaldehyde, and 5-hydroxymethylfuran [27].

**Figure 1:**
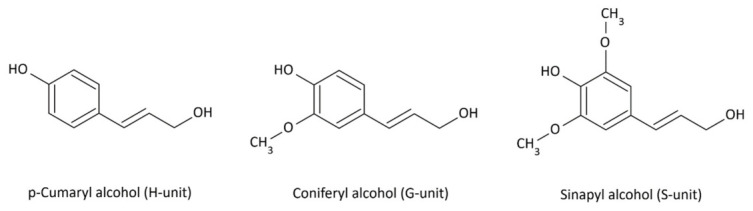
The primary structural units of lignin.

Lignin is a branched biopolymer composed primarily of the monomers p-coumaryl, coniferyl, and sinapyl alcohols (Figure 1). It is the third most abundant component in wood after cellulose and hemicellulose. In softwood, it occurs at concentrations ranging from 23 to 33%, whereas in hardwood it varies from 16 to 25% [13]. This material is more thermally resistant than hemicellulose and cellulose. Lignin degrades gradually and begins to decompose at temperatures above 250 °C. Maximum degradation rates are observed at temperatures between 350 and 450 °C [28]. Complete decomposition of this polymer occurs at 500 °C [20]. During heating, complex depolymerization, condensation, and oxidation reactions occur. The resulting products depend not only on the processing temperature but also on the polymer’s chemical composition, i.e., the ratio of p-coumaryl alcohol (H-unit), coniferyl alcohol (G-unit), and sinapyl alcohol (S-unit) units. Individual units of this biopolymer are linked by carbon-carbon (C-C), ester, and ether bonds [29].

**Figure 2:**
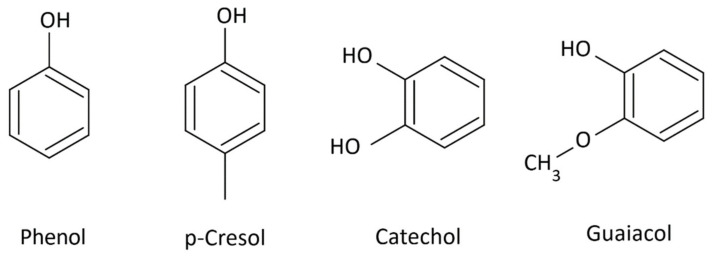
Examples of phenolic compounds produced during the pyrolysis of lignins.

**Figure 3:**
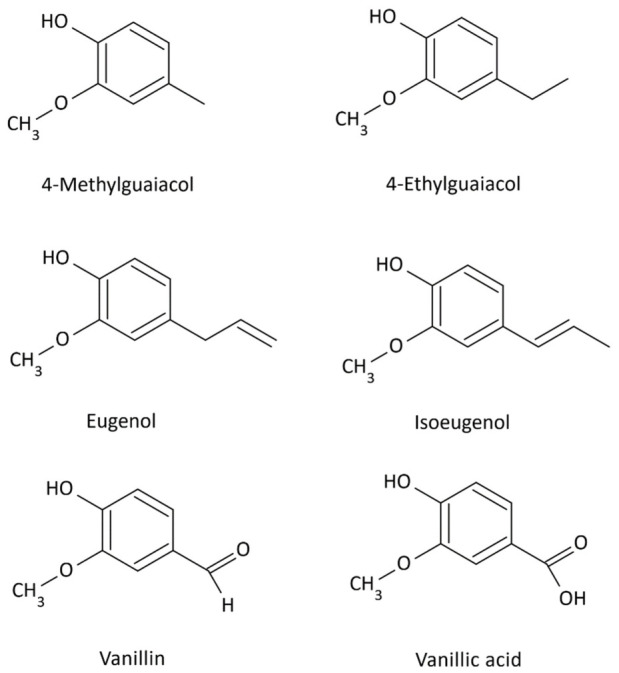
Examples of guaiacol derivatives produced during the pyrolysis of lignins.

**Figure 4:**
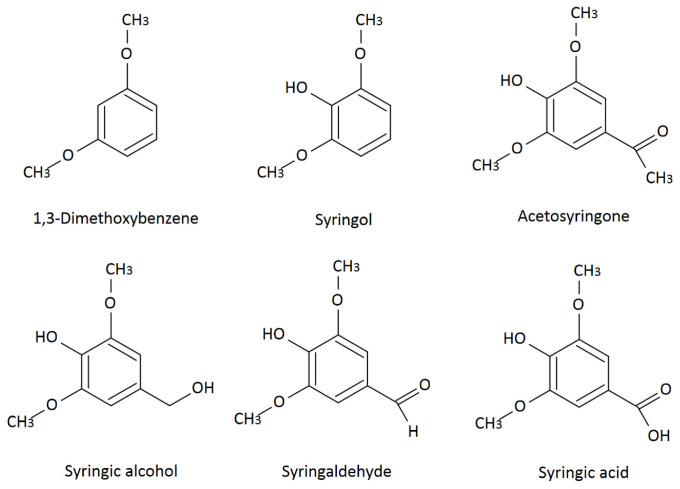
1,3-Dimethoxybenzene derivatives formed during the pyrolysis of lignins.

The least susceptible to degradation are the p-coumaryl alcohol groups; they do not contain methoxy groups. Increasing the number of methoxy groups in the units makes the coniferyl alcohol, which includes one methoxy group, pyrolyze more readily. In contrast, sinapyl units, which contain two methoxy groups, are most susceptible to degradation. Thermal decomposition of lignin produces numerous volatile low-molecular-weight phenols: p-cresol, catechol, guaiacol, 4-methylguaiacol, 4-ethylguaiacol, eugenol, isoeugenol, vanillin, vanillic acid, syringaldehyde (Figure 2, Figure 3 and Figure 4), and lactones [29,30].

Low-molecular-weight compounds, particularly those containing methoxy groups (Figure 2 and Figure 3), formed during pyrolysis undergo numerous secondary reactions, including repolymerization, dealkylation, demethoxylation, and isomerization [29,30,31].

**Table 1:** The main products of thermal decomposition of wood components.

Wood Component, Decomposition Temperature	Structural Unit of a Chemical Compound	Main Thermal Decomposition Products	References
Hemicelluloses 150–260 °C	sugars: glucose, xylose, mannose, galactose sugar acids: glucuronic and galacturonic acid	furfural, hydroxymethylfurfural, formic, acetic, and propionic acid, formaldehyde, acetaldehyde, hydroxyacetaldehyde, γ-butyrolactone, and other low molecular weight lactones	[23,24,32]
Lignin 200–500 °C	p-coumaryl alcohol (H-unit)	phenol, methylphenols, alkylphenols, p-hydroxybenzaldehyde, p-hydroxybenzoic acid, p-hydroxyphenol	[29,30,31]
	coniferyl alcohol (G-unit)	guaiacol, methylguaiacol, ethyl guaiacol, vinyl guaiacol, vanillin, vanillic acid	
	sinapyl alcohol (S-unit)	syringol, methylsyringol, ethylsyringol, vinylsyringol, acetosyringone, propylsyringone, syringaldehyde, syringic acid	
Cellulose 260–450 °C	glucose	aldehydes, furans, acids, α- pyranone, levoglucosenone, phenol, benzene, ethylbenzene, p-xylene, o-cresol, styrene	[27,33]
Sugars 180–450 °C	glucose, fructose, sucrose, xylose, arabinose, galactose	aldehydes, furans, maltol, acids	[11,33,34]
Polyphenols	ellagic and gallic acid derivatives	pyrogallol, catechol, resorcinol, phenol	[35,36,37,38,39,40]
Acids (lipids)	fatty acids	aldehydes and ketones, lactones, short-chain acids	[41,42,43,44,45,46,47]

During intense barrel firing, local temperatures can reach 500 °C, causing charring of organic matter, particularly where it contacts the heat source. However, because wood is a poor conductor of heat, the temperature inside the fired wood is usually lower. The temperature of American oak (*Q. alba*) samples heated with a butane torch did not exceed 150–200 °C [34]. Simple sugars contained in wood and compounds formed during the breakdown of complex carbohydrates also undergo numerous pyrolysis reactions. Depending on oxygen availability and concentration, they primarily produce furans (furfural and HMF), aldehydes, and ketones, with small amounts of acids, esters, and harmful substances such as benzene, phenol, and crotonaldehyde [33]. The concentration of wood pyrolysis products is not uniform. For HMF, furfural, and vanillin, the concentration in the outer (2 mm) layer of toasted oak staves was one hundred to several dozen times higher than the concentration in the wood layer at a depth of 10 mm. Burning the staves also causes physical changes in the wood, creating cracks that facilitate evaporation and the loss of volatile compounds such as lactones. The concentration of oak lactones is lower at the surface and several times higher in deeper layers of the wood [34].

Tannins, naturally present in wood, also undergo numerous reactions at high temperatures [37,38]. The course of the process depends on the composition of the atmosphere, primarily the presence of oxygen. The oxidation and condensation processes of polyphenolic compounds occur at low temperatures during storage, transport, and mechanical processing of wood [35]. As temperature increases, oxidation reactions accelerate. Seasoned oak wood contains only small amounts of volatile phenols, primarily eugenol, and traces of phenolic aldehydes. Pyrolysis produces numerous mono- and dimethoxylated phenols and high levels of benzoic and cinnamic aldehydes [48]. Heat treatment of oak wood (120–250 °C) causes a reduction in the amount of ellagitannins and a simultaneous increase in ellagic acid [49].

During wood combustion, oxygen availability is limited, so we primarily observe pyrolysis, i.e., the thermal degradation of organic substances in the absence of oxygen. At temperatures above 200 °C, ester bonds break down. Decarboxylation removes the carboxyl group from phenolic acids, releasing gaseous products: carbon dioxide and carbon monoxide, resulting in the formation of alcohols and phenols [40].

**Figure 5:**
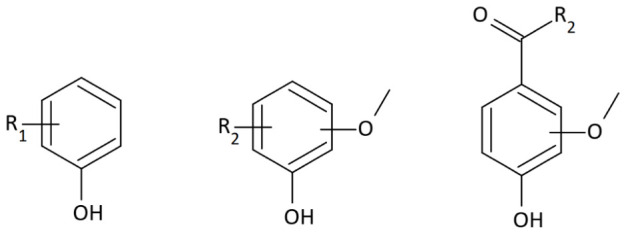
Phenol derivatives identified in wood pyrolysis products. R_1_: -alkyl or -OH, R_2_: -alkyl.

Dehydration of molecules leads to an increase in the number of unsaturated compounds (double bonds are formed), the aromatic rings decompose, and numerous phenols are formed: phenol, catechol (1,2-dihydroxybenzene), pyrogallol (1,2,3-trihydroxybenzene), and their multiple derivatives (Figure 5). Fragmentation of polyphenols also occurs at high temperatures, which leads to the formation of aldehydes, ketones, alcohols, and numerous other compounds [36,39]. During heating, a change in the elemental composition is observed. The share of hydrogen and oxygen decreases, while the percentage of carbon increases. Wood loses water and, in addition to carbon dioxide and other inorganic gases, such as ammonia and numerous volatile organic compounds, releases simple hydrocarbons, including methane, ethane, and acetylene [50]. Thermal treatment of wood also leads to the formation of numerous aromatic hydrocarbons. Many of these compounds are harmful and carcinogenic, or suspected of being carcinogenic, e.g., toluene (from cresol, phenol, and benzoic acid), xylene, dimethylbenzene, and 1,3,5-trimethylbenzene [40,41].

**Figure 6:**
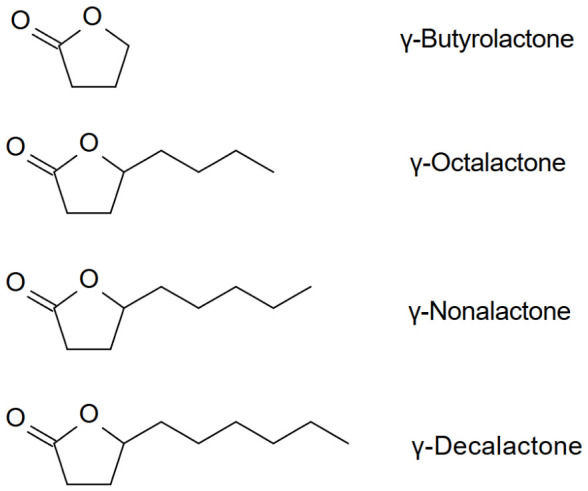
Lactones present in oak wood.

Aliphatic acids are also present in wood. They occur in bound forms, such as lipids (esters of glycerol and higher fatty acids), and in free form. Alkyl acids and hydroxy acids are also formed during the degradation of lignin side chains. These hydroxy acids then yield lactones such as γ-butyrolactone, γ-octalactone, γ-nonalactone, and γ-decalactone (Figure 6), as well as their derivatives, with aromas of coconut, peach, apricot, nutty, roasted, or woody [41,42,43,44,45,46,47]. Alcohol (H-unit), coniferyl alcohol (G-unit), and sinapyl alcohol (S-unit) units. Individual units of this biopolymer are linked by carbon-carbon (C-C), ester, and ether bonds [29].

**Figure 7:**
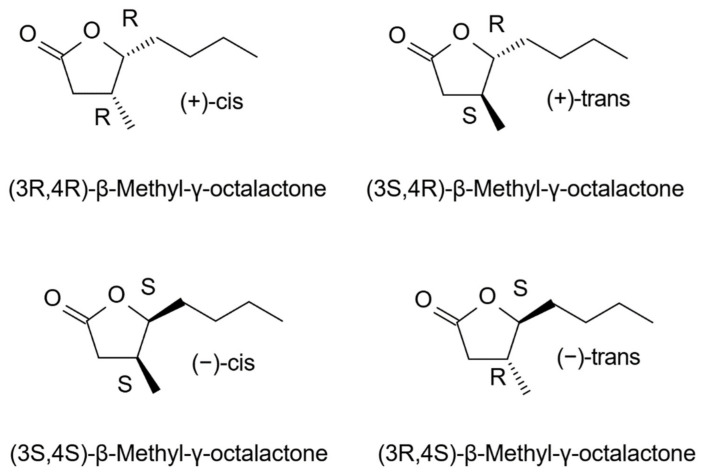
Four β-methyl-γ-octalactone isomers.

γ-Octalactone derivatives: *cis*-whisky lactone (quercus lactone, *cis*-3-methyl-4-octanolide, (3S,4S)-β-methyl-γ-octalactone, (3S,4S)-5-butyl-4-methyldihydrofuran-2-(3H)-one) and *trans*-whisky lactone ((3S,4R)-β-methyl-γ-octalactone), γ-nonalactone, and γ-decalactone are characterized by very low sensory detection thresholds [43,51,52]. Of the four stereoisomers of β-methyl-γ-octalactone (Figure 7), only the 3S,4S (*cis*) and 3S,4R (*trans*) forms are present in oak wood [53]. The concentration of whiskey lactones in medium- and high-toasted oak wood chips of French and American origin ranges from 13 to 58 mg/kg [54].

Another lactone extracted from wood during the storage of beverages in barrels is γ-butyrolactone (GBL) [54], which is classified as a depressant. This substance acts on the central nervous system [55]. Its concentration in wines ranges from 0.5 to 0.8 mg/L, which is much lower than the perception threshold, which is approximately 20 mg/L [3] and below any measurable effect on the human body, because clinical studies have shown that mild side effects such as short-term amnesia, hypotonia and euphoria can be expected after taking GHB doses below 10 mg/kg [55].

**Figure 8:**
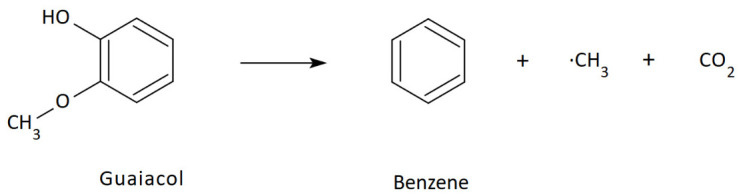
Thermal decomposition of guaiacol to benzene, methyl radicals, and carbon dioxide.

Incomplete combustion of wood can produce substances potentially harmful to human health. Some of these substances exhibit carcinogenic and mutagenic properties. Simple aromatic hydrocarbons such as benzene, toluene, ethylbenzene, xylene, styrene, naphthalene, and their numerous alkyl, vinyl, methoxy, and phenyl derivatives have been found in wood pyrolysis products [40,56]. These substances are potentially harmful to health, especially with long-term exposure, and benzene is a known carcinogen [57]. During the pyrolysis of lignin, a variety of volatile alkylated phenols and keto-alkoxyphenols are produced [58,59]. Benzene is formed by thermal decarboxylation of benzoic acid and cleavage of the methoxy group from the guaiacol (Figure 8) and syringol units of lignins [59].

**Figure 9:**
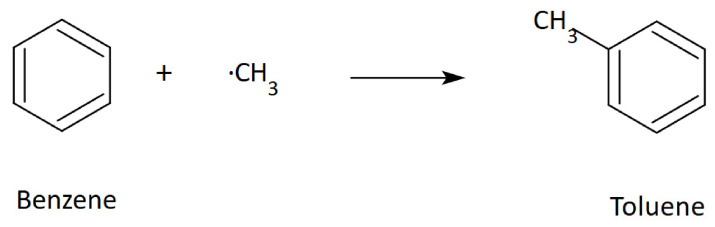
Synthesis of toluene by the reaction of benzene with methyl radicals.

In addition to gases (carbon dioxide and carbon monoxide), the reaction produces extremely reactive methyl radicals, which can react with benzene and its derivatives (Figure 9), forming toluene and cresols in secondary reactions [59].

In the smoke produced during the combustion of oak wood, apart from benzene and toluene, other aromatic compounds were detected, such as styrene, ethylbenzene, xylene, 1,2,4-trimethylbenzene, 1,3,5-trimethylbenzene, aliphatic derivatives (acrolein, 1,3-butadiene, acetonitrile), and chloride derivatives (chloromethane, trichlorotrifluoroethane, and vinyl chloride) [60].

**Figure 10:**
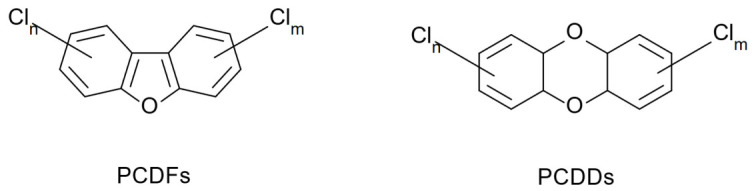
Hazardous to health wood pyrolysis products: polychlorinated dibenzofurans (PCDFs), 1 ≤ (n + m) ≤ 8 and polychlorinated dibenzo-p-dioxins (PCDDs) n ≤ 4, m ≤ 4.

Under specific conditions, wood combustion and pyrolysis also produce higher-molecular-weight hydrocarbons. Of particular note are polycyclic aromatic hydrocarbons (PAHs), polychlorinated dibenzo-p-dioxins (PCDDs), and polychlorinated dibenzofurans (PCDFs) (Figure 10) [61,62,63].

**Figure 11:**
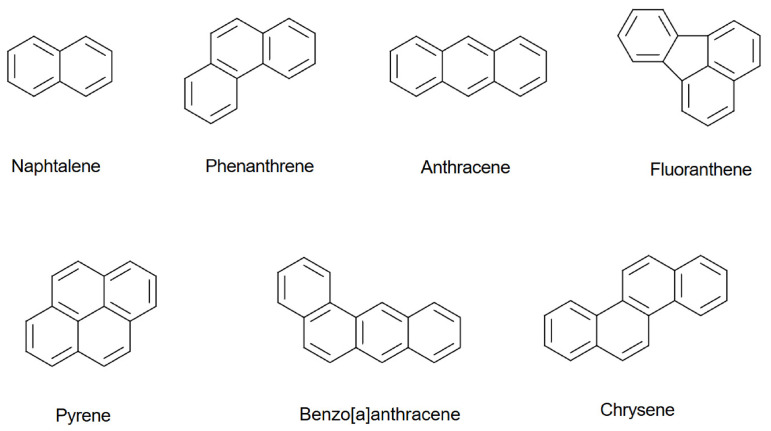
Polycyclic aromatic hydrocarbons detected in oak wood pyrolysis products.

Polycyclic aromatic hydrocarbons are produced during the combustion of wood, fossil fuels, biomass, waste, and industrial processes. They are present in tobacco smoke, water, air, and food. Multiple routes of exposure may increase the adverse effects of these compounds on human health. In food, PAHs are formed during high-temperature processing, such as roasting coffee beans and roasting barley malt for beer and whisky production, during grilling, direct drying, smoking, or by adding smoke agents [64,65,66]. Polycyclic aromatic hydrocarbons are hydrophobic and poorly soluble in water. When charred oak barrels are used to store alcoholic beverages, the ethanol present in the beverage, as an excellent solvent, increases the risk of PAHs migrating into it [67]. Naphthalene, phenanthrene, anthracene, fluoranthene, pyrene, benzo(a)anthracene, and chrysene (Figure 11) were identified during the pyrolysis of oak wood [40].

Wood chips used in the production of alcoholic beverages contained very small amounts of PAHs. The compounds were mainly low- and medium-molecular-weight compounds, ranging from 0.96 to 15.1 ng/g of wood. Some samples detected the carcinogenic compounds benzo[a]pyrene and benz[a]anthracene [68]. Due to their low solubility, only a small amount of aromatic hydrocarbons in wood migrates into the beverage produced. For comparison, Scotch malts used in whisky production contain many times (two or three orders of magnitude) higher concentrations of PAHs than toasted wood [69].

During wood combustion, potentially hazardous polychlorinated dibenzo-p-dioxins (PCDDs) and polychlorinated dibenzofurans (PCDFs) can be formed [70,71,72,73]. However, there are no reports on the influence of pyrolysis of the surface of oak staves on the formation of PCDDs and PCDFs and their migration into barrel-aged beverages.

It should be noted that the main component of alcoholic beverages, ethanol, is classified by the International Agency for Research on Cancer (IARC) as a human carcinogen (Group 1). In the body, ethanol is oxidized to acetaldehyde by alcohol dehydrogenase (ADH). Acetaldehyde reacts with DNA bases to form adducts, which are critical in carcinogenesis because they can cause miscoding, leading to mutations and loss of normal growth-control mechanisms [74]. Other substances classified by the IARC as carcinogenic compounds, such as formaldehyde, acrylamide, arsenic, lead, cadmium, and benzene, may be formed in alcoholic beverages or extracted from wood. Epidemiological studies have shown that ethanol is the main oncogenic component in alcoholic beverages [75]. However, the synergistic effect of carcinogens produced during pyrolysis and ethanol cannot be ruled out, especially in tobacco smokers [76].

## 5. The Importance of Wood-Derived Compounds in the Production of Alcoholic Beverages

Among the several hundred compounds derived from natural wood and those formed during its toasting, several groups can be distinguished that are most important for the aroma and flavor of alcoholic beverages. These include volatile and non-volatile steroids, esters, alcohols, terpenes, terpenoids, lactones, and volatile phenols [77,78,79]. Table 2 presents examples of compounds from the aforementioned groups, along with their sensory detection thresholds.

### 5.1. Wines

Classic winemaking consists of several stages. In the first stage, the fruit is sorted to eliminate moldy or rotten fruit. It is then crushed to disrupt the tissues and reduce pulp particle size. This creates a looser structure, facilitating pressing and increasing yield. The next stage of vinification, crucial for red wines, is maceration of the pulp. This process increases the leaching of aromatic compounds and tannins, improving juice extraction efficiency. Sulphiting of the pulp is also employed at this stage to provide microbiological and oxidative stabilization. Typically, wine-specific noble yeast strains are also added at this stage. After pre-treatment and maceration, the crushed fruit pulp is pressed to obtain must. Alcoholic fermentation is a key process in winemaking, in which yeast breaks down the sugars in the must, producing ethyl alcohol, CO_2_, and several other substances known as by-products: glycerol, succinic acid, lactic acid, acetic acid, higher alcohols, aldehydes, and other compounds present in minimal amounts but crucial to the taste and aroma of wine (including esters and terpenoids). Many yeast species and strains are used for fermentation, differing in their tolerance to alcohol, temperature, SO_2_, pH, and sugar levels, as well as in their efficiency in converting sugars to ethyl alcohol and in the types and amounts of fermentation by-products produced. The type of yeast used directly influences the taste, aroma, and character of the finished wine [90,91,92,93]. Young wines don’t yet exhibit quality characteristics; these develop during aging. Aging is a crucial stage in achieving high-quality wines, during which a series of physical and chemical changes occur. Aging increases the wines’ stability, but above all, develops their complex aromas [79,94].

Aroma is one of the most essential characteristics shaping wine quality, and its profile can comprise more than 1000 volatile compounds. The aroma of wines depends on several factors, such as grape variety and cultivation methods, climate, vintage, vinification methods, and, above all, maturation conditions [95,96,97]. Volatile compounds derived from fruit constitute the primary aroma of wines. Some of these are bound as non-volatile glycosides, which are aroma precursors. During fermentation, enzymes derived from yeast, lactic acid bacteria, or enzymatic preparations hydrolyze the glycosidic bonds of precursors, thereby releasing aroma-forming compounds (secondary aromas). Furthermore, many of the compounds that constitute wine’s aroma are formed during fermentation, for example, from amino acids. The final aromas, particularly important in the wine’s bouquet, known as tertiary aromas, develop during aging in oak barrels or in contact with wood [97,98].

The volatile compounds responsible for shaping wine aromas belong to various groups. These include esters, fatty acids, aldehydes, ketones, terpenes, alcohols, lactones, and volatile phenols, with concentrations ranging from mg/L to μg/L or even ng/L. Most of these compounds enhance the aromatic profile of wines, whereas others can have adverse effects, depending on their concentration and the perceived odor threshold. Only compounds with an odor active value (OAV) greater than one can shape the aroma of wines [97,99,100,101].

Barrel aging is considered the most crucial step in shaping wine aromas. This primarily applies to red wines, but white wines aged in contact with wood also acquire a unique aroma. This technique is still widely used. The resulting aroma profile is influenced by many factors, including the type of wood used to make the barrels, their degree of toasting, the length of time the wine spends in the barrel, and the surface area of contact between the wine and the wood (barrel volume) [12,102,103,104,105].

Table 3 presents the concentrations of selected aroma-shaping compounds in wines aged in barrels made from various wood species. The grape varieties used and the degree of barrel toasting are taken into account. Most researchers’ experiments (Table 3) were conducted in medium-toasted barrels; however, the effects of toasting degree and barrel aging time on the profiles of aroma-shaping compounds will be discussed in greater detail later in this paper.

The most common wood species used for barrel aging was French oak. Most experiments involved a 12-month barrel aging period. It can be seen (Table 3) that furfural and hydroxymethylfurfural, as well as lactones and syringaldehyde, migrate significantly from French oak barrels to the wines, regardless of the grape variety from which the wine was made. However, the grape variety often plays a significant role in the rate of compound transfer from wood to wine. Wines from Cabernet Sauvignon, Sauvignon blanc, and Chardonnay tended to contain higher concentrations of compounds from French oak than wines from Mencía, Merlot, and Tempranillo [88,109,111]. It should be noted, however, that even within the same grape variety, wood type, and wine maturation time, significant differences in the concentrations of these compounds are observed. For example, in Tempranillo wines, significant differences were observed in the concentrations of compounds migrating from French oak [109,111], and, for furfural, 5-methylfurfural, and HMF, the differences were up to 10-fold. The degree of toasting was very important for the migration of aroma compounds from the barrels, though not always clear-cut. Sauvignon blanc wines aged in lightly toasted French oak barrels contained lower concentrations of most of the analyzed compounds than those aged in medium-toasted barrels (Table 3). Higher concentrations of lactones were noted in wines matured in lightly toasted barrels [88]. Similar trends were observed in Cabernet Sauvignon wines, but in this case, wines from lightly toasted barrels contained higher levels of eugenol, syringol, acetovanillone, and maltol than those from medium-toasted barrels [109,115]. Different trends were observed during the maturation of Chardonnay wines [88].

In this case, lightly charred French oak barrels were the source of higher levels of furfural and its derivatives, guaiacol and its derivatives, and vanillin and syringaldehyde. Mencía and Merlot wines aged in Spanish oak barrels contained significantly fewer compounds migrating from the wood than those matured in French oak barrels (Table 3) [95,107,109]. However, lactone concentrations were higher in wines aged in these barrels. For Tempranillo wines, more valuable compounds migrated from the barrels when Spanish oak was used than when French oak was used [109,111]. The levels of most compounds derived from French and Spanish oak were comparable in Cabernet Sauvignon wine [109,115]. The concentration of guaiacol in wines aged in oak barrels ranged from approximately 4.6 µg/L [88] to 120 µg/L [112], and eugenol from 7.78 µg/L [109] to 116 µg/L [111]. It’s noticeable that, in many cases, French oak barrels contribute significantly fewer of these aromatic compounds to wine than American or Spanish oak. The migration of compounds from American oak barrels was comparable to that from Spanish oak wines and was dependent on the grape variety [108,111].

Wines matured in chestnut barrels contained significantly lower concentrations of most wood-derived compounds compared to wines matured in oak barrels. Differences were particularly noticeable for lactones and vanillin. However, higher concentrations of guaiacol and its derivatives were observed in wines matured in chestnut barrels than in wines matured in oak barrels. Higher amounts of wood-derived compounds were also found in Tempranillo wines matured in toasted and untoasted chestnut barrels [106,107,109,110,111]. It should be noted that cherry and ash wood barrels, apart from syringaldehyde, do not contribute significant amounts of aroma-forming compounds to the wines matured in them, especially compared to wines matured in oak barrels. Wines matured in barrels made of cherry, acacia, and ash wood did not contain lactones at all [106].

From the point of view of the sensory perception of wines, not only is the content of aroma-shaping compounds important, but also their sensory detection threshold. HMF and 5-methylfurfural migrated most from oak, chestnut, and acacia wood. However, it should be emphasized that these compounds have a relatively high olfactory threshold (15–100 mg/L), producing vanilla, toast, and caramel flavors [89]. Significant amounts of lactones, particularly whiskey lactone in the yew conformation, were observed in most oak-aged wines, regardless of the grape variety used. However, these values varied significantly across studies, ranging from 53 µg/L [107] to 1370 µg/L [112]. Lactones contribute coconut, woody, and vanilla aromas to wine. These compounds are particularly important for wine aromas due to their low olfactory thresholds, with the cis form at 20–46 µg/L [89]. Compounds present at relatively low concentrations are also important from an aroma perspective, but because of their very low olfactory thresholds, they significantly alter the sensory impression. Examples of such volatile components include guaiacol and eugenol, with olfactory thresholds of 9.5 and 6 µg/L, respectively. Guaiacol gives wines a smoky, sweet, and medicinal aroma, while eugenol gives wines a clove, honey, spicy, and cinnamon aroma [89]. The concentration of guaiacol in wines aged in oak barrels was approximately from 4.6 µg/L [88] to 120 µg/L [112], and eugenol from 7.78 µg/L [109] to 116 µg/L [111]. It’s noticeable that, in many cases, French oak barrels contribute significantly fewer of these aromatic compounds to wine than American or Spanish oak. It’s also worth noting that chestnut barrels are a good source of guaiacol and eugenol, regardless of the type of wine matured in them (Tempranillo, Syrah) [106,110]. One of the compounds that contributes to the smoky, burned, and woody aromas characteristic of toasted wood in wines is syringol. It occurs in wines aged in oak barrels at varying concentrations, ranging from 27 to 374 µg/L [97,115]. It is also worth noting that its concentration was much lower in wines from Spanish oak barrels than in wines from French or American oak barrels. Syringol, on the other hand, is present in large quantities in wines matured in chestnut-wood barrels [106,110].

**Figure 12:**
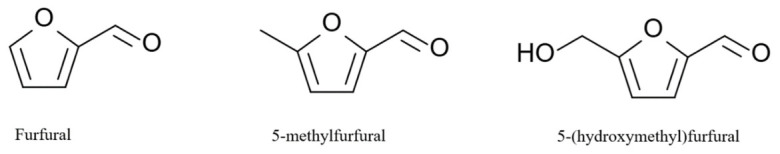
Thermal decomposition products of pentoses and hexoses.

The profile of compounds shaping the aroma of wines stored in barrels significantly depends on the duration of maturation [113] reported that maturation of Syrah wine in new American white oak (*Q. alba*) barrels over 9 months reduced the amount of furfural and 5-methylfurfural (Figure 12) from 3920 to 736 µg/L and from 613 to 435 µg/L, respectively.

**Figure 13:**
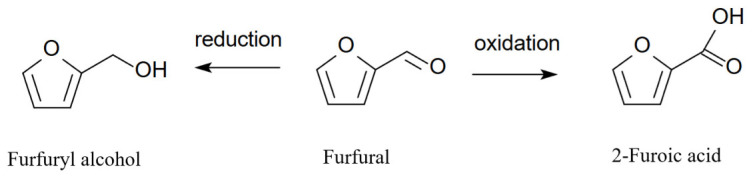
Furfural transformations in wine products.

The content of these compounds in previously used barrels was even lower. However, the concentration of lactones, particularly in the *cis* conformation, increased significantly, from 259 µg/L to 1332 µg/L after 9 months of aging. The number of compounds responsible for the phenolic, smoky, and medicinal aromas of the wines: 4-ethylguaiacol and 4-ethylphenol, also increased significantly up to 6 months of aging, while longer aging led to a slow increase in the concentrations of these compounds, regardless of whether the barrels were new or previously used. Similar trends were observed for vanillin [113]. These observations were partially confirmed in another work [112]. Analyzing wines aged in barrels for 18 months, the authors observed similar trends in lactone concentration, but the differences between months 6 and 18 were less pronounced. Guaiacol, eugenol, 4-ethylguaiacol, and 4-ethylphenol did not change their concentrations in the wines throughout the experiment. However, the results for furfural and 5-methylfurfural were different—no significant changes in concentrations were observed throughout the experiment [112]. Furfural, depending on the conditions, can be reduced in the presence of yeast to furfuryl alcohol [116] or oxidized to 2-furoic acid (Figure 13) [117,118].

In another study [114], wines were aged for 18 months in French oak barrels. The authors demonstrated a significant increase in the concentration of furfural and its derivatives up to the 12th month of the experiment, followed by a dramatic decrease (over five-fold). The levels of vanillin, guaiacol, and eugenol in the wines remained similar throughout the experiment. The concentration of lactones increased up to the 12th month of aging and remained similar thereafter, but their concentration was significantly lower than in the Bautista-Ortín et al. study [113].

The stabilization of the volatile phenol concentration in Tempranillo wines aged for 12 months in American oak (*Q. alba*) barrels was demonstrated in another study [103]. The authors report that the concentrations of furanic compounds (furfural, 5-methylfurfural) decreased significantly between the 6th and 12th months of maturation, confirming earlier studies. However, the results of these studies are inconsistent with the changes in lactone concentrations. Feng et al. (2023) reported that the concentration of both *cis*- and *trans*-lactone did not change significantly between 6 and 12 months of aging in wine [103]. A significant increase in the amount of furanic compounds during 12 months of wine aging in oak barrels was demonstrated in another study [97]. The concentration of most volatile phenols also increased throughout the maturation period, with the exception of 4-ethylguaiacol, 4-vinylphenol, and 4-vinylguaiacol, whose amounts remained stable or decreased slightly after 3 months of maturation. As in other studies cited earlier, extending maturation time increases lactone levels in wine [97].

The degree of toasting of the wood is of great importance for the profile of compounds shaping the aroma of wine during barrel maturation. Navarro et al. [119] examined changes in the content of compounds derived from barrels with different degrees of toasting made of American oak (*Q. alba*) and French oak (*Q. petraea*) for 12 months in white (Macabeo) and red (Cabernet Sauvignon) wines. They found that, regardless of wine type, the levels of furanic compounds and volatile phenols increased with increasing toasting. However, the content of lactones decreased significantly with increasing toasting. Vanillin concentrations were highest in moderately toasted barrels and lowest in heavily toasted barrels. Levels of all volatile compounds were substantially lower in wines aged in one-year-old barrels than in new barrels. American oak released significantly higher amounts of lactones, especially the *cis* isomer, than French oak at all toasting levels and in new and one-year-old barrels [119]. Other experiments [120] involved maturing oloroso Sherry wine in medium-toothed and heavily toasted American oak (*Q. alba*), French oak (*Q. robur*), Spanish oak (*Q. pyrenaica*), and chestnut (*C. sativa*) barrels. The authors showed that as the degree of toasting increased, the amount of furfural increased slightly in each barrel type, while 5-methylfurfural remained stable, and even decreased in the chestnut barrel. The authors [120] also report that greater toasting increases the concentrations of guaiacol, ethylguaiacol, and eugenol in wines. Chestnut wood did not contain lactones, but in wines aged in oak barrels, particularly French oak, a significant increase in lactones was observed with increasing toasting. These results are inconsistent with those presented in the previously cited work [119]. The authors of other studies have shown that the degree of French oak barrel toasting and 12-month maturation of Sauvignon blanc wines increase the concentrations of most wood-derived compounds, including furfurals (furfural, 5-methylfurfural), whiskylactones, ethylphenols, eugenols, and vanillins. The only compounds whose concentrations were lower in wines matured in barrels with a high degree of toasting were vinylphenols. The same experiments, but with Chardonnay wine, yielded different results. Higher amounts of furfural and 5-methylfurfural were observed in the more heavily toasted barrels, but the concentration of HMF was lower. Lactone concentrations were also lower in wines aged in more heavily toasted barrels. The degree of barrel toasting did not significantly change the concentration of eugenols in Chardonnay wines. The remaining compounds analyzed were similar to those found in Sauvignon blanc wines [119].

Barrel aging, primarily in oak, is a classic technique for enriching wines with wood-derived aromas, typically with oak toasted to varying degrees. However, this technique is expensive. Barrels are usually made from the most valuable parts of the tree trunk. Furthermore, wines aged in new barrels and those aged in reused barrels release varying amounts of compounds that shape their aromas. In response to the high costs of traditional barrel aging and the impacts of deforestation, new techniques have emerged that involve aging wine with wood chips. The use of oak chips to enhance the sensory characteristics of wines has been documented in France as early as the 19th century. In some countries, such as Australia, the USA, South Africa, and Chile, this practice has been used for many years. In recent years, their use in Europe has been regulated by the EU Commission directives [121,122,123].

Wood fragments, such as chips, staves, or cubes, are often less expensive than barrel production and can be sourced from less valuable parts of trees, primarily from waste generated by the broader wood industry. From a technological perspective, wood fragments offer winemakers valuable opportunities for product diversification. However, their proper use depends on several factors, including the dimensions and toasting of the wood pieces, the duration of contact with the wine, and the complex interactions between the wine and the yeast. It’s important to note that the surface area of contact between the wine and the wood chips is significantly larger than in a barrel, so a small amount of wood chips is used, and the maturation time is considerably shorter than in classic barrel maturation [123,124].

The emergence of new aging technology using wood chips has prompted extensive research into their impact on wine quality [113,123,125,126,127,128].

The experiments [123] compared the profile of compounds derived from various wood fragments: staves (2.2 × 2.2 × 90 cm), cubes (5 × 5 × 1.8 cm), and irregularly sized chips. Staves and cubes were added at 300 g/hL, and chips at 150 g/hL, and all wood types were moderately toasted. The results showed that the concentrations of syringaldehyde, vanillin, *trans*-whiskey lactone, and methyl guaiacol were significantly higher in wines aged with wood chips. In some cases, these values were even 4–5 times higher. In most cases, the smallest amounts of aroma-forming compounds were transferred from staves. However, in the case of furfural, wines from cubes contained the highest levels of this compound, whereas wines with wood chips contained none. Similar trends were observed for methyl-5-furfural. The levels of guaiacol and its derivatives were identical across all wood particles used [123]. Other experiments [129] on the aging of Bobal wines, including a 1-week aging with oak chips at 3 and 6 g/L, showed a greater increase in the concentration of substances migrating into the beverage than in the previously cited study. The results obtained were 5- to 7-fold higher than those reported by Asproudi et al. [123], despite the chips doses being only twice as high and the aging times being 12 times shorter (one week and 3 months, respectively). Furthermore, based on their experience, the authors demonstrated that doubling the number of chips did not double the proportion of compounds shaping the wines’ aromas. The results were approximately 60% higher when twice as many chips were used [129]. Different results were obtained in another study by [130]. Experiments on the aging of Tempranillo wines showed that the concentration of most wood-derived compounds increased most rapidly during the first month. The amounts of vanillin, syringaldehyde, furfural, and 5-methylfurfural were significantly higher in wines aged with staves than with wood chips. However, wood chips yielded higher lactone concentrations, particularly in the *trans* conformation.

Laqui-Estaña et al. [126] analyzed the extraction of phenolic compounds from barrels, wood chips, and staves in Carménère and Cabernet Sauvignon wines. They showed that during the initial days of wine maturation, significantly higher amounts of these compounds migrated from wood chips and staves than from barrels. Maximum concentrations of these compounds in the wines were reached after approximately one month of aging with wood chips and staves. Longer aging (over six months) equalized the concentrations of wines aged in barrels and those aged with wood particles. These results demonstrate that using wood chips can significantly shorten the maturation time of wines in contact with wood and reduce production costs.

In studies on Chardonnay wine aged for 3 months in barrels or with the addition of oak chips, it was shown that many compounds migrating from wood to wine (e.g., 2-methoxy-4-vinylphenol) reached maximum values after 1 month of the experiment, and extending the maturation time did not significantly affect their level [128]. Barrel-aged wines showed lower concentrations of these compounds, even after three times longer aging time. Furthermore, the authors demonstrated that moderately toasted wood chips contribute more aroma-forming compounds to wines than lightly toasted chips. Similar results for Muscat Ottonel white wines were obtained in another study [127].

The authors report that the extraction rate of volatile phenols from wood chips is faster and more efficient than from barrels. Furthermore, it was shown that volatile phenols are transferred into wines from toasted wood chips more efficiently than from untoasted wood chips. In a study by [125], based on analysis of whiskey lactones, volatile phenols (guaiacol, 4-methylguaiacol, eugenol, isoeugenol), vanillin, and acetovanillone, wood aromas are transferred more quickly and efficiently into wine when wood chips are added during fermentation rather than during aging.

As with barrels, the temperature at which chips are toasted is critical [121]. The authors aged Merlot wines for extended periods, and Table 4 presents selected maturation times for wines with oak chips of varying toast levels. As shown, in all cases where chips were used, higher levels of aroma-forming compounds were observed than in the control (the sample without added chips). In wines aged in the presence of medium-toasted oak chips, higher levels of most aroma-forming compounds were observed compared to wines aged with lightly and heavily toasted oak chips. However, lactone concentrations were higher in wines containing lightly toasted chips. Heavy toasting reduced the amounts of all aroma-forming compounds in the wines (Table 4).

Similar results were reported in another study [122] for most volatile compounds identified in wood chips, which shape the aroma of Agiorgitiko wines.

The authors demonstrated that the concentration of extractable compounds from wood increased with increasing wood chip quantity, but this increase was not proportional. However, unlike the previously cited work [121], the present authors demonstrated that guaiacol concentration increased with increasing experimental duration and was absent in samples containing lightly toasted wood chips.

Martínez-Gil et al. [131] analyzed the effect of oak-chips origin on the volatile compound profile of Carménère wines. Four oak species were used: French and Romanian oak (*Q. petraea*), American oak (*Q. alba*), and Colombian oak (*Q. humboldtii*). The results were surprising—they showed enormous variability depending on the tree’s origin. The content of furfural and its derivatives, vanillin, and lactones was highest in wines aged with American and Romanian oak chips. Colombian oak was rich in guaiacol, eugenol, and their derivatives, and the contents of guaiacol and isoeugenol were 4–10 times higher than in the other chips tested. However, Colombian oak chips contributed almost no sensory valuable lactones to the wines.

**Figure 14:**
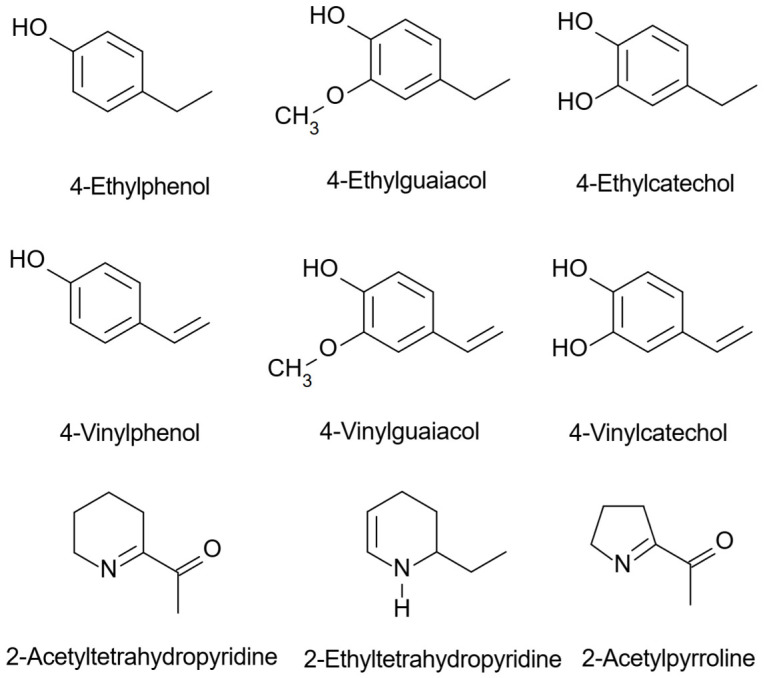
Volatile metabolites of *Dekkera* and *Brettanomyces* yeasts.

Many of the compounds derived from wood, such as acetic acid, polyphenols (vanillin, syringaldehyde, ferulic, and coumaric acids), furan compounds (furfural and 5-hydroxymethyl-furfural), terpenes, and terpenoids, also have antimicrobial properties [132,133,134,135,136,137]. Furan compounds can inhibit the activities of the enzymes alcohol dehydrogenase, aldehyde dehydrogenase, and pyruvate dehydrogenase, thereby inhibiting alcoholic fermentation [138]. Yeasts reduce reactive carbonyl compounds (acetaldehyde, furfural, HMF, glycolaldehyde, vanillin) to the corresponding alcohols (ethanol, furfuryl alcohol, ethylene glycol, vanillin alcohol) [139]. In wines aged in wood, the extracted antimicrobial compounds may contribute to greater product stability. On the other hand, the porous structure of wood facilitates secondary contamination of wine by *Dekkera* and *Brettanomyces* yeasts. This yeast can cause spoilage in wine, producing aromas such as horse, rancid, sweaty saddle, barnyard, smoke, or medicinal notes due to the production of volatile phenols: 4-vinylphenol, 4-ethylphenol, 4-vinylguaiacol, 4-ethylguaiacol, 4-ethylcatechol, and 4-vinylcatechol (Figure 14). Low molecular weight aliphatic acids (isovaleric acid, isobutyric acid, and 2-methylbutyric acid) and heterocyclic derivatives (2-acetyltetrahydropyridine, 2-ethyltetrahydropyridine, and 2-acetylpyrroline) are also detected in infected wines [140,141,142,143,144,145,146,147,148].

The risk of *Dekkera* and *Brettanomyces* yeast contamination increases with repeated barrel use, so wood chips or staves (wood powders, chips, staves, or cubes) are a safer alternative [123,149,150]. Thermal treatment of wood (toasting) not only affects sensory aspects (color, aroma) but also limits the number of microorganisms released into the wine. Chemical disinfection methods, such as adding sulfur dioxide or ozonating the wood, are an alternative [151].

The volatile compound profile of wood can be adjusted by using a specific wood species and its degree of toasting. In the case of barrels, the number of times they are used will be crucial. New, toasted barrels contribute significantly higher amounts of aroma-forming compounds, and repeated use can translate into lower extraction of compounds from the wood, even at concentrations below the sensory threshold. This problem does not occur with wood chips and staves, as new chips are prepared each time.

The use of moderately toasted barrels and wood fragments (wood chips, staves) results in higher levels of most aroma-forming compounds. Lighter toasting does not sufficiently break down lignin and hemicelluloses, limiting their migration into the wine. Over-toasting contributes to the degradation of the most valuable aroma components, reducing their levels to levels even below the sensory threshold.

Using oak chips and aging wines for approximately 3 months produces similar results to aging wines in barrels with the same degree of toasting for approximately 1 year. When wood chips are added to wine, the concentration of lactones peaks after 1 month of aging and then decreases. This trend is not observed in barrels. The usual dose of wood chips (3–6 g/L) may be insufficient to achieve trans-lactone concentrations above their sensory threshold. The concentration of other extractable components from wood usually exceeds the threshold values.

This knowledge enables winemakers to control the toasting method and aging time to extract the desired levels of compounds that shape the woody aroma of wines. This will depend on the type of wine and the sensory effect consumers expect. Using wood chips for aging wines also allows producers to use several wood species simultaneously, enabling even more precise control over the qualitative and quantitative profiles of the compounds that shape the wines’ aromas.

### 5.2. Beer

The basic ingredients of beer are barley malt, water, hops, and yeast. The crushed malt is mashed, which activates specific groups of enzymes derived from the malt, primarily α- and β-amylases, proteases, and β-glucanases. Starch is hydrolyzed mainly to maltose, the principal fermentable sugar in brewing. Hops are then added to the resulting wort, which is then brewed to extract bittering and aromatic compounds, sterilize it, and obtain the proper extract of the base wort. The wort is then cooled to fermentation temperature, depending on the yeast used: bottom-fermenting yeast to approximately 5–10 °C, and top-fermenting yeast to approximately 10–15 °C. A key stage in beer production is the maturation of young beer, during which it develops its characteristic aroma and flavor. Typically, this process takes place in stainless steel tanks. However, intense competition among producers, particularly the emergence and development of numerous craft breweries producing distinctive beers, and consumer awareness are driving the search for new, interesting, and attractive flavor combinations. One approach is to use wood for maturing beers in the form of barrels or wood fragments, such as wood chips or staves. The use of timber in beer maturation is not new. Before the construction of stainless-steel tanks, oak barrels were used to store and transport beer. Some beers, such as Belgian beers and porters, still use oak barrels to enhance their flavor and aroma [152,153,154,155,156]. However, the use of wood in beer maturation is uncommon and occurs much less frequently than in wines and distillates.

In a study by Bossaert et al. [157], three types of beers were aged in oak barrels: stout (alcohol content 9.64%), top-fermented blond beer with a lower alcohol content of 3.12%, and top-fermented blond beer (alcohol content 8.88%). Among the compounds shaping the aroma associated with wood, the concentrations of eugenol and oak lactones *cis*-β-methyl-γ-octalactone and *trans*-β-methyl-γ-octalactone were found to increase over time in all beers. However, their concentrations were higher in beers with higher ethanol levels. Vanillin content, on the other hand, was highest in light beers. In the lighter beer, its concentration increased significantly in the first 12 weeks of aging. The greatest increase in vanillin concentration was observed in the light, strong beer (818 μg/L after 38 weeks of aging). The vanillin concentration in stout remained uniform throughout the barrel-aging period.

The amounts of furfural and 5-methylfurfural remained constant during the maturation of the beers, and their amounts were higher in beers matured in new oak, compared to those already used. The concentration of 4-vinylguaiacol was higher in light beer, whereas monophenolic compounds such as 4-hydroxybenzaldehyde, guaiacol, 4-ethylphenol, and 4-ethylguaiacol were present at the highest levels in stout beer, and maturation for more than 12 weeks significantly increased their concentrations in the samples [157].

Belgian Dark Strong Ale 21°P was matured in port wine barrels for 16 weeks [158]. The authors found that the concentration of *cis*-β-methyl-γ-octalactone increased with maturation time, whereas *trans*-oak lactone was higher after 11 weeks of maturation and then decreased significantly (from 50.5 to 8.4 μg/L). Lactones were absent in the fresh beer. The levels of volatile phenols, which were absent in the fresh beer, increased during maturation, but the differences between 11 and 16 weeks were not statistically significant.

In another study, a top-fermented blond beer with 10.31% alcohol was aged in oak and acacia barrels [154]. The concentration of wood-derived aromatic compounds increased during the maturation process, particularly in oak barrels. The concentrations of eugenol, *cis*-β-methyl-γ-octalactone, and *trans*-β-methyl-γ-octalactone increased significantly in oak barrels, by 5-80-fold, whereas in acacia barrels they increased by only a few percent. A similar trend was observed for vanillin, whose concentration increased from 24.29 to 1376.60 μg/L in beers matured in oak barrels and to 472.53 μg/L in beers matured in acacia barrels after 38 weeks of maturation. In contrast, the concentration of total polyphenols doubled in acacia barrels, while a slight decrease in concentration was observed in oak barrels.

The degree of wood particle toasting during beer maturation also significantly influences the migration of aroma compounds from wood into the beverage [152]. Experiments were conducted on beers aged with oak cubes of varying degrees of toasting. It was shown that contact with wood increased the concentrations of gallic acid, 5-HMF, and furfural compared to the control (beer stored in bottles). The addition of heavily toasted cubes significantly increased the amounts of gallic acid and 5-HMF. However, it was noted that light toasting of the oak cubes resulted in high furfural concentrations (approximately 10-fold) in the beers. An undesirable effect was also noted: the presence of oak cubes during beer maturation adversely affected sensory evaluation, regardless of the degree of toasting [152].

The impact of wood-chips type (American and French oak) and different degrees of toasting on the aromatic compound profile of Belgian beer (8.3% alcohol by volume) was assessed in another study [159]. The authors showed that syringaldehyde concentrations increased in all cases, but most significantly in heavily toasted chips. Similar results were obtained for acetosyringone, vanillin, and acetovanillone, particularly in American oak. Concentrations of these compounds increased throughout the experiment in heavily toasted American oak chips. Still, they reached a maximum after 32 days of aging in medium-toasted American oak chips and heavily toasted French oak chips. Concentrations of guaiacol and 4-ethylguaiacol increased as the beers matured after most oak-chips types were added. Eugenol, thymol, and salicylaldehyde reached their highest concentrations in medium-toasted oak chips. In contrast, concentrations of 4-vinylguaiacol and 4-ethylphenol remained constant throughout the experiment, regardless of oak type and degree of toast [159]. The same authors, continuing their research, demonstrated that as the oak-chips dose used for beer maturation increased from 2 to 15 g/L, the concentrations of most monophenols studied increased in a nearly-linear manner. A very slow increase was observed for eugenol, whereas the concentration of 4-vinylguaiacol was relatively uniform and little affected by the wood chip dose. The authors also noted that the extraction of monophenols from wood was favored by the beer’s low pH and high alcohol content [160].

The effect of wood chips from different wood species on the extraction of compounds that shape the wood aroma of beers was investigated in another study [161]. Selected results are presented in Table 5.

The analyzed wood chips exhibit similar syringaldehyde release into the beers. Balsam wood is a good source of vanillin and furfural, while amburana chips contain 5-hydroxymethylfurfural. Horse chestnut chips released significant amounts of most compounds tested into the beers. Oak chips generally contributed lower concentrations of the analyzed compounds, except 5-hydroxymethylfurfural.

As a result of 30-day maturation of beers with wood chips, a reduction in the concentration of many aliphatic alcohols was demonstrated. This phenomenon was particularly evident in lager beers. This may be related to the adsorption of alcohols by wood. Interestingly, however, nonanol can be extracted from wood, as observed in macerated ales and lagers. Maceration with wood chips reduced ester levels, especially ethyl esters. This phenomenon was more evident in ales than in lagers or porters. Maceration of beers with oak chips also significantly reduced terpene levels, likely due to adsorption in the wood [162].

Other studies [163] showed that American oak chips significantly increased the concentrations of certain polyphenols compared with French oak chips. The authors also showed that, for beers, the share of many phenolic compounds depends primarily on the type of malt rather than on the wood in which it is aged. In another work [164], it was noted that craft breweries have greater opportunities to implement new methods for maturing beers in contact with various types of wood, even those not yet used in brewing. It has been shown that the use of wood in brewing is very important in the production of sour beers. Lactic acid bacteria multiply better in contact with the porous structure of wood and more easily produce metabolites responsible for the aroma characteristic of sour beers [165].

Beer maturation differs significantly from that of wine and spirits. First and foremost, the duration of maceration is crucial, and in the case of beer, this is considerably shorter. Wine and spirits are aged for periods ranging from several months to several decades, whereas beer is typically aged for just a few weeks. However, even a short maturation period in contact with wood can significantly alter the beverage’s composition by extracting compounds from toasted wood. In the case of beers, many different types of wood can be used, often exotic varieties, which may be of particular interest to craft breweries wishing to produce new, unique beverages. Experiments with fermenting beers in contact with wood, rather than just ageing them, may also be of interest, but this approach carries a risk of microbial contamination.

It should also be noted that many of the compounds that shape the composition and aroma of beers and are characteristic of wood can be formed in malt, and it is the malt itself that may be their primary source. Therefore, although these beers have low alcohol content and are aged in barrels for only a short time, they contain significant concentrations of compounds such as HMF, furfural, and vanillin.

### 5.3. Spirits

Spirits comprise an extensive range of beverages, primarily made from various grains and other starchy raw materials, as well as fruit and molasses. They are divided into clear and flavored vodkas. Clear vodkas are mixtures of carefully purified ethanol and water in appropriate proportions. Flavored vodkas are a broad category of products that retain the sensory notes of the raw materials from which they are made and owe their sensory qualities to long-term maturation, typically using wood. The production of spirits involves preparing a mash by adding water and appropriate enzymes (primarily α- and glucoamylases) to ground grains, which break down starch into fermentable sugars, mainly glucose, and then fermenting the mixture with yeast. Fruit mashes are not subjected to enzymatic treatment. Fermentation then takes place, yielding a mash with an alcohol content of 10–15% by volume. After fermentation, the mash is distilled, but the resulting distillate is usually harsh and pungent, unsuitable for consumption. Therefore, the next stage is maturation, during which the flavor and aroma harmonize, producing numerous compounds responsible for the smell of these vodkas, primarily esters. This is the most crucial stage in the production of flavored vodkas. Maturation can last from several months to several decades. An essential element of maturing flavored vodkas is their contact with wood, which extracts wood-derived compounds into the beverage, imparting the desired aroma and flavor. Because flavored vodkas contain 40–50% alcohol by volume and the maturation process usually lasts several years, the extraction of compounds derived from the wood can be crucial to their quality [166,167,168,169,170]. The high-alcohol beverages most commonly associated with maturation in wooden contact, primarily in barrels, are whisky and brandy.

#### 5.3.1. Whisky (Or Whiskey)

Whiskey originates from Ireland, and the first records of its production date back to 1405. Today, it is produced worldwide, particularly in the United Kingdom, the United States, Canada, Japan, India, China, France, and Thailand. Whiskey is made by distilling a mash of malted grains, primarily barley (*Hordeum polystichum*), but also corn (*Zea mays*), rye (*Secale montanum*), and wheat (*Triticum vulgare*). Aging distillates in wooden barrels is a traditional and mandatory process in whisky production. The physicochemical and sensory changes that occur during aging play a key role in shaping whisky quality and are determined by the type of barrel used and the aging method [167,168,171,172,173,174].

As with other alcoholic beverages, the composition of the barrel wood significantly affects the profile of compounds that shape the whisky’s aroma, which varies with the type of oak used, the wood’s initial treatment, and the number of times the barrel is used. In addition to barrel type, other factors, such as filling level and storage conditions, influence the maturation process. Higher temperatures accelerate evaporation, increase extract levels, and some reaction rates, but do not necessarily yield a more mature product [175].

Compounds extracted from wood for whisky represent various chemical groups. Of particular importance and characteristic for oak wood are *cis*- and *trans*-lactones, furfurans (e.g., furfural, 5-methylfurfural, HMF), phenolic aldehydes (vanillin, acetovanilone, syringaldehyde), and volatile phenols (4-vinylguaiacol, guaiacol, 4-methylguaiacol, 4-ethylguaiacol, eugenol) [176,177]. Their amounts in whisky can vary significantly, and the concentration ranges of selected compounds derived from oak wood are presented in Table 6.

It has also been shown [177] that the origin of the wood has a significant impact on the aromatic profile of whisky. The authors used *Quercus mongolica* from China, *Q. alba* from America, and French *Q. robur* in their experiments. They showed that whisky matured in contact with Chinese oak had lower furan levels (2–3 times) than whisky from other barrels. *trans*-Whisky lactone was highest in samples matured in French oak and lowest in Chinese oak (over 10-fold), whereas whisky matured in American oak was rich in *cis*-whisky lactone. The level of phenolic aldehydes was less dependent on the oak species used, with French oak being the best source. Similar trends were observed for guaiacol derivatives, and eugenol concentration was not reliant on the type of wood used [177].

In another paper [178], the authors reported that the content of whiskey lactones in whiskey matured in *Q. mongolica* and *Q. alba* barrels was similar after 20 years of maturation. However, maturation for 40 years increased the amount of lactones in whiskey from *Q. mongolica* barrels more than 5-fold compared to whiskeys from *Q. alba* barrels.

The toasting method also significantly affected the profile of compounds that shape the whisky’s aroma. Increasing the degree of toasting increased the concentrations of pyrroles (with a nutty, sweet aroma) and volatile compounds derived from thermodegraded polysaccharides (e.g., furfural, 5-methylfurfural, HMF). Higher toasting temperatures and times, on the other hand, reduced pyrazine concentrations, producing a popcorn-like roasted aroma. The amounts of lactones, on the other hand, were highest in whisky matured with moderately toasted wood. Higher temperatures reduced the concentrations of these valuable whisky aroma components [49,179,180].

Some types of whisky are matured in barrels previously used for maturing wine. The use of sherry barrels increases the level of tannins and sugars in the whisky [175]. It has also been shown that three years of maturation in a sherry cask yields more furfural and its derivatives and phenolic aldehydes compared to dynamic maturation in a solera system [174].

Industrial whiskey maturation is carried out exclusively in barrels. However, there are reports of attempts to use wood particles to mature whiskey in the laboratory. The authors [178] matured whiskey in 1-L tanks filled with American and Chinese oak cubes. They showed that after 12 months of experimentation, whisky with Chinese oak cubes released almost twice as much furfural and its derivatives, as well as vanillin. They also observed nearly twice the lactone concentration in whisky with Chinese oak cubes. Laboratory experiments using heavily roasted oak (*Q. alba*) cubes to mature whisky revealed that char depth, rather than charring temperature, had a greater impact on the resulting whisky profile during aging.

Other studies [181] demonstrated the possibility of determining geographical origin and authenticating barrel maturation time based on non-volatile profiles using complex and costly FT-ICR-MS and chemometric analyses. The authors analyzed compounds formed during fermentation, distillation, and maturation. Evaluating compounds derived from wood enabled the determination of realistic maturation times in oak barrels, ranging from 6 to over 18 years.

#### 5.3.2. Brandy

Brandy is an alcoholic beverage made from various fruits, most commonly grapes. The most famous grape brandy is cognac, produced exclusively in the French department of Charentaise. The fruit undergoes alcoholic fermentation using noble yeasts or spontaneous fermentation. The resulting wine is distilled to an alcohol content of less than 94.8% by volume and, in addition to ethanol, contains volatile fermentation products that contribute to the brandy’s aroma and serve as precursors of other compounds that also contribute to the beverage’s aroma. After distillation, brandy is colorless and has an alcohol content of 60–70% by volume. The beverage is then matured, most often in barrels. Then, the aroma harmonizes: new compounds form, shaping the brandy’s aroma (primarily esters), and wood components migrate into the brandy, building the drink’s final aroma. Woody’s notes are typical and characteristic of Brandy. Their quantity and quality, like those of other alcoholic beverages discussed earlier, depend on many factors, including the type of wood, the surface area of contact between the distillate and the wood, the duration of the process, and the degree of wood toasting [85,168,182,183,184,185].

Fiches et al. [186] studied commercial grape brandies aged in oak barrels for different periods. They found that the profile of volatile compounds derived from oak wood varied with maturation time. The concentrations of whiskey lactones (*cis* and *trans*), phenolic acids (e.g., vanillic acid, syringic acid), and vanillin were three times higher in whiskeys aged for over 8 years compared to those aged for two years. The syringaldehyde level increased from 2 mg/L to approximately 5 mg/L, whereas the sinapaldehyde level decreased with increasing maturation time. A slight increase in the amount of 5-methylfurfural and HMF was also observed [186].

Similar results to those presented by [186] were obtained in another study [187]. The authors also demonstrated that extending the time brandy was stored in barrels increased the concentration of most volatile compounds derived from wood. However, contrary to the results of the previously cited study, the concentration of sinapaldehyde was uniform across brandies aged for up to 10 years, after which it declined significantly. It was also shown [188] that repeated use of barrels, both wine and brandy, significantly reduced the amount of most volatile compounds derived from wood. The highest amounts of these compounds are observed after brandy has matured in new barrels.

Barrel aging is a traditional technique that has been used for hundreds of years. However, the search for cheaper, more efficient methods to age brandy is prompting producers to explore alternatives to barrels. One such option is the use of wooden chips or staves. Their use significantly shortens the brandy’s aging time by increasing the surface area of contact between the beverage and the wood. It was shown [189] that two years of maturing brandy in barrels and with wooden staves (91 × 5 × 1.8 cm) with the same degree of toasting had different effects on the levels of wood-derived compounds. The authors reported that most of the wood’s volatile compound characteristics were transferred to brandy in greater quantities from the barrels rather than from the staves. Examples of such compounds include *cis*- and *trans*-lactones of whisky, 5-methylfurfural, HMF, eugenol, and vanillin. Brandies matured with staves contained higher concentrations of syringol and acetovanillone. Similar trends were observed in another study, which used identical staves (91 × 5 × 1.8 cm) for brandy maturation [190]. The authors also reported that micro-oxygenation applied to stainless steel barrels with staves significantly increased the concentrations of wood-derived compounds in the brandy, sometimes exceeding those observed in oak barrels. However, the contact surface area of the staves is small relative to that of the wood chips. Experiments on apple brandy maturation using wood chips and barrels have shown that wood chips are a better source of volatile aroma compounds [191]. These values were 3 to 6 times higher for wood chips than for barrels.

It has been shown [192] that aging time increases the concentrations of all tested wood-derived compounds in distillates. The authors examined time spans ranging from less than 3 years to 8–10 years and observed that the amounts of lactones, HMF, and vanillin increased sixfold with increasing experiment duration, while syringaldehyde and guaiacol increased three- and fourfold, respectively. Still, furfural increased 17-fold, and eugenol nearly fortyfold. Other studies [193] indicate that aging brandy with wood chips at temperatures below 0 °C increases the concentrations of specific compounds compared with ageing at room temperature. These include HMF, furfural, and lactones.

The type of wood used for maturation also plays a significant role in shaping the volatile compound profile [194]. American oak wood is a rich source of lactones, especially *cis*-lactones. French oak contained slightly less, whereas Spanish oak, chestnut, and cherry wood contained no lactones. The wood types studied are similar sources of furfural and its derivatives (5-methylfurfural and HMF). All oak species studied contribute significantly more vanillin and syringaldehyde to beverages (4–10 times) than chestnut and cherry. Brandy matured in 16-L barrels made of different wood types and with varying degrees of toasting was studied in another study [195]. Contrary to the results of the previously cited publication, the authors report that Spanish oak and chestnut wood are better sources of furfural and its derivatives than American and French oak. Similar results were obtained for vanillin and sinapaldehyde. Syringaldehyde levels in brandy aged in French oak barrels were the lowest among those studied. They also demonstrated that medium-toasted oak barrels yield lower concentrations of vanillin and syringaldehyde than heavily toasted barrels. Furfural derivatives in American and French oak barrels were found in higher concentrations in heavily toasted barrels than in medium toasted barrels. In both cases, however, heavily toasted chestnut barrels were a poorer source of the compounds described (vanillin, syringaldehyde, furfural, and HMF) [195].

#### 5.3.3. Cachaça

The main and most recognizable barrel-aged distillates are whiskeys and brandies made from various fruit species. However, cachaça is also barrel-aged, and the aging method influences its quality. Cachaça is an alcoholic beverage produced exclusively in Brazil, made from sugarcane. It can be aged in barrels made from various types of wood, like other distilled beverages. The choice of wood type has a different impact on color, flavor, and aroma [196]. It has been shown [197] that cachaça aged in oak barrels contains more furfural and HMF than that aged in American oak barrels. The remaining wood-derived compounds tested, e.g., vanillin and syringaldehyde, were at similar levels during 24 months of aging. Authors [197] also showed that heavily toasted barrels release about twice as many wood-derived compounds into cachaça as moderately toasted ones.

In another work [198], it was reported that compounds shaping the aroma of cachaça and originating from American oak occur at almost twice the concentration as those from European oak.

In other studies [199], French oak chips with varying degrees of toasting were used to mature cachaça. It was shown that all the analyzed compounds (including 5-hydroxymethylfurfural, furfural, vanillin, vanillic acid, sinapaldehyde, and syringaldehyde) originating from the chips and shaping cachaça quality migrated in the highest amounts when medium-roasted chips were used. Cachaça made with lightly and heavily toasted chips contained significantly lower amounts of the tested components.

Barrels made of wood typical of Brazil, e.g., amburana (*Amburana cearensis*), jatoba (*Hymenaea carbouril*), balsam (*Myroxylon peruiferum*), and peroba (*Paratecoma peroba*), may be a source of different concentrations of compounds migrating into cachaça during maturation than oak [200]. The authors paid particular attention to jatoba wood because cachaça matured with it had more than twice as much furfural as cachaça matured in oak barrels. As with other alcoholic beverages, maturing cachaça in reused barrels results in a significantly lower share of compounds derived from barrel wood [201].

Distillates are characterized by a significantly higher alcohol content than the previously discussed wines and beers. Furthermore, these alcoholic beverages are aged in barrels, primarily oak, for significantly longer than wines. For most distillates (whisky, brandy, rum), this process takes at least 3 years, but sometimes as long as 40 years. This results in relatively much higher levels of these compounds than in wines. The values for lactones, vanillin, syringaldehyde, and acetovanilone can be as much as 20–30 times higher. The smallest discrepancies occur for guaiacol and its derivatives, and HMF.

The profile of compounds released from barrels during distillate maturation, as with wines, is highly dependent on the number of times they are used. New barrels contribute significantly more of these compounds to the distillates. The degree of barrel toasting is an equally important factor. The greatest amounts of aroma-forming components are usually released from barrels with a medium toasting. Legal regulations and cooperage capabilities limit the types of wood used to make barrels and mature distillates. However, within the range of wood types used and the possibility of varying degrees of toasting, distillate producers can regulate the quantitative and qualitative composition of compounds derived from the wood to a certain extent. The planned maturation time of the distillates is also important. By combining different types of wood and varying degrees of toasting, distillers can control the maturation process.

The limited laboratory research on the use of wood particles may provide a foundation for further industrial-scale experiments. Using wood chips to mature distillates may be another avenue to improve control of the maturation process for these spirits.

## 6. Conclusions

Barrels are still used for the production and storage of alcoholic beverages. Wood in contact with the beverage significantly affects its quality. Chemical compounds naturally occurring in wood, such as polyphenols, resins, fats, and waxes, are extracted during ethanol fermentation and the maturation of alcoholic beverages. Another large group of substances migrating into alcoholic beverages from wood is pyrolysis products, formed during barrel firing. Currently, barrels are increasingly being replaced by steel, concrete, or ceramic tanks. To achieve results similar to those with oak barrels, the tanks are filled with thermally treated wood (staves or chips) added to the beverage during maturation, and micro-oxygenation is employed.

Insoluble biopolymers contained in wood, as a result of thermal degradation and secondary reactions, decompose into numerous substances, many of which have a very low sensory threshold. Volatile polyphenols, aldehydes, and lactones formed during pyrolysis impart a unique character to alcoholic beverages that come into contact with burned wood. The chemical composition and number of substances migrating from wood depend mainly on the alcoholic beverage produced. Higher alcohol concentrations enable the extraction of substances that are insoluble in water but highly soluble in ethanol.

The complex chemical composition of alcoholic beverages and the presence of microflora, particularly in wines, contribute to numerous secondary reactions between components leached from wood and the components of the maturing beverages. This contributes to the extraordinary complexity of the chemical reactions and the large group of products formed in beverages. This work focuses primarily on compounds that influence beverage aroma. However, a large group of chemical compounds remains incompletely understood, primarily because of their low concentrations in beverages and the challenges of quantitative analysis. At high temperatures, partial combustion and pyrolysis of wood can potentially produce aromatic hydrocarbons, posing a potential threat to human health. It’s crucial to understand that wood pyrolysis conditions must be monitored.

Treating wood at excessively high temperatures and processing contaminated wood, especially with substances containing chlorine or heavy metals, carries the risk of producing substances harmful to health. It should be noted that the main ingredient in alcoholic beverages, ethanol, is classified as a human carcinogen. Other substances classified by the IARC as carcinogenic, such as acetaldehyde, formaldehyde, benzene, and heavy metals, may be present in beverages or be extracted from wood.

The development of modern chromatographic and spectroscopic techniques has significantly advanced our understanding of aroma components, but certain groups of compounds, particularly potentially harmful substances such as PAHs, PCDFs, and PCDDs, have yet to be tested in a representative sample of alcoholic beverages. A broader analysis of these substances could contribute to a better understanding of the relationship between cancer and alcohol consumption.

## Figures and Tables

**Table 2:** The main compounds derived from wood and shaping the aroma of alcoholic beverages [42,45,80,81,82,83,84,85,86,87,88,89].

Common Name	IUPAC Name	Olfactory Threshold	Aroma Notes
**Volatile phenols**			
4-Ethylguaiacol	4-ethyl-2-methoxyphenol	47 µg/L	phenolic, smoked, leather
4-Methylguaiacol	4-methyl-2-methoxyphenol	20 µg/L	spicy, phenolic, light green
4-Vinylguaiacol	4-vinyl-2-methoxyphenol	40 µg/L	clove
Eugenol	2-methoxy-4-(prop-2-enyl)phenol	6 µg/L	clove, honey, spicy, cinnamon
Guaiacol	2-methoxyphenol	9.5 µg/L	smoke, sweet, medicine
Isoeugenol	2-methoxy-4-propenylphenol	6 µg/L	floral, clove, woody
Syringol	2,6-dimethoxyphenol	570 µg/L	smoke, burned, wood
**Lactones**			
*cis*-β-Methyl-γ-octalactone	(3S,4S)-5-butyl-4-methyldihydro-2-(3H)-furanone	20–46 µg/L	coconut, woody, vanilla
*trans*-β-Methyl-γ-octalactone	(3S,4R)-5-butyl-4-methyldihydro-2-(3H)-furanone	140–370 µg/L	coconut, woody, vanilla
**Heterocyclic compounds**			
5-Hydroxy-methylfurfural	5-hydroxymethyl-2-furaldehyde	100 mg/L	caramel
5-Methylfurfural	5-methyl-2-furancarboxaldehyde	16 mg/L	almond, caramel, burnt, sugar
Furfural	furan-2-carbaldehyde	15 mg/L	bread, almond, sweet
Maltol	3-hydroxy-2-methyl-4H-pyran-4-one	5 mg/L	honey, toasty, caramel
**Phenolic aldehydes/Phenyl ketones**			
Acetovanillone	1-(4-hydroxy-3-methoxyphenyl)ethanone	1 mg/L	vanilla
Syringaldehyde	4-hydroxy-3,5-dimethoxybenzaldehyde	50 mg/L	vanilla
Vanillin	4-hydroxy-3-methoxybenzaldehyde	1 mg/L	vanilla

**Table 3:** Concentrations of selected wood-derived compounds in wines aged in barrels made of different types of wood [88,97,106,107,108,109,110,111,112,113,114,115].

Wine Type (Maturation Time mth) Toasting Level *	Compounds Concentration (µg/L)														
	Furfural	5-Methylfurfural	HMF	Maltol	*trans*-Whiskylactone	*cis*-Whiskylactone	Guaiacol	4-Methylguaiacol	4-Ethylguaiacol	4-Vinylguaiacol	Eugenol	Syringol	Vanillin	Syringaldehyde	Acetovanillone
**French oak (*Q. petraea*)**															
Cabernet Sauvignon (12) M	836	289	114	111	49.5	142	19.6	7.2	na	na	7.8	73.5	62	23.3	11.4
Cabernet Sauvignon (12) L	300.7	71.1	na	331.6	80.2	116.8	40.9	22.3	2.7	89.1	29.4	373.9	44.5	52.2	97.6
Chardonnay (Spain) (12) M	1966	105	2039	na	160	281	4.6	6.5	na	257	20	na	187	791	24
Chardonnay (Spain) (12) L	8995	885	6973	na	108	187	17	21	na	294	19	na	611	3177	46
Mencía (12) M	110	51.4	1229	92	245	611	27.2	24.2	31.5	73.5	22.7	na	289	743	102
Merlot (12) M	739	109	43.3	132	33.8	152	28.9	15.9	na	na	25.2	74.3	42.6	23	19.6
Sangiovese (Italy) (12) M	na	na	na	na	na	99.9	53	na	89.9	57.6	28.3	na	99.1	na	na
Sauvignon blanc (Spain) (12) M	13837	1111	13149	na	162	276	27	17	na	219	31	na	678	6467	47
Sauvignon blanc (Spain) (12) L	891	39	1444	na	180	353	9.3	5.8	na	249	30	na	173	850	28
Somontano (Spain) Syrah (12) M	39.8	842	703	142	99.4	577	43.8	31.5	24.2	20.8	101	178	408	1305	62.4
Tempranillo (12) M	155	46.7	44.6	150	25	154	28.1	9.8	na	na	32.7	72.5	48.4	9	22.3
Tempranillo (12) M	1378	869	986	114	305	529	36.3	25.6	5.6	409	35.7	na	466	1495	195
Tempranillo (60%), Cabernet Sauvignon (20%), Garnacha (20%) (12) M	771	135	na	na	73	79	8.8	0.06	87	na	20	na	89	312	114
Tempranillo (63%), Cabernet Sauvignon (20%), Merlot (17%) (12) M	127	439	na	119	412	629	11.8	na	na	na	91.8	na	216	60.5	na
**Spanish oak (*Q. petraea*)**															
Cabernet Sauvignon (12) M	851	276	100	92.4	24	214	11.2	2.7	na	na	17.1	34.1	45.7	5.4	7.3
Mencía (12) M	190.5	70.8	na	na	98.9	199	14.4	5.8	7.6	9	9.5	27.6	29.5	na	22.5
Mencía (12) M	134	93.5	1704	111	222	1011	42.1	36.9	43.7	93.1	84.2	na	342	553	121
Merlot (12) M	55	119	51.7	105	36.3	322	14.5	2.7	na	na	20.5	37.6	43.6	3.4	11.4
Tempranillo (12) M	165	74.6	77.9	150	44.6	253	34.3	11.8	na	na	25	81.7	42.8	12.2	14.3
Tempranillo (12) M	3575	2477	3141	282	231	1096	76.1	21.9	5.3	315	116	na	574	1748	203
**American oak (*Q. alba*)**															
– (12) M	5330	250	na	na	210	1370	120	na	450	na	100	na	na	na	na
Mencía (12) M	187	41.8	2094	197	236	1313	40.9	32.5	24.9	109	51.9	na	540	1378	153
Monastrell (9) M	739	435	na	na	80	1332	na	na	64	na	na	na	431	na	na
Tempranillo (12) M	1869	1543	1193	168	150	1083	41.5	21.9	5	242	63.3	na	482	1495	175
Tempranillo (63%), Cabernet Sauvignon (20%), Merlot (17%) (12) M	232	488	na	135	152	845	22.4	na	na	na	81.5	na	165	66.3	na
**Chestnut (*Castanea sativa*)**															
Sangiovese (Italy) (12) M	na	na	na	na	na	43.6	54.3		79.5	37.7	36.3	na	20	na	na
Somontano (Spain) Syrah (12) M	509	241	689	125	21.3	21.2	59.3	51.5	49.1	23.9	118	153	456	1189	92.4
Tempranillo (6) N	24.4	64.3	3.3	2.4	na	na	35.1	24.5	133.9	41.4	139.4	285.4	156.7	na	135.8
Tempranillo (6) M	28.5	184	6.2	3.3	na	na	35	29.8	123.5	37.7	162.1	242.1	260.7	na	155.1
**Cherry (*Prunus cerasus* L.)**															
Somontano (Spain) Syrah (12) M	101	31.8	145	133	nd	nd	42.8	24.6	73.3	23.1	10.5	169	304	1877	75.1
**Acacia (*Acacia* Mill.)**															
Somontano (Spain) Syrah (12) M	238	450	248	300	nd	nd	59.8	14.1	19.9	25.7	19.3	217	233	768	61.3
**Ash (*Fraxinus excelsior* L.)**															
Somontano (Spain) Syrah (12) M	66.2	57.8	339	354	nd	nd	75.1	34.8	91.5	9.9	12.8	198	696	1090	111

* The barrel wood was subjected to different toasting conditions: N—Non-toasted wood, L—Lightly toasted, M—Medium toasted, nd—not detected, na—not analyzed.

**Table 4:** Evolution of oak volatile concentration (µg/L) in wine during maturation [121].

Compound	Control		Light Toast		Medium Toast		Heavy Toast	
	1 Month	3 Months	1 Month	3 Months	1 Month	3 Months	1 Month	3 Months
5-Methylfurfural	1.12	1.12	23.5	69.26	30.44	72.41	5.9	12.52
*cis*-Whiskylactone	3.52	3.24	43.5	104.8	13.19	23.93	5.83	7.98
Eugenol + isoeugenol	1.4	0.8	4.25	4.65	2.39	2.16	1.84	1.09
Furfural	84.25	67.9	132.4	339.2	478.57	682	104.9	129.95
Guaiacol	42.95	7.04	26.29	8.76	72.32	20.6	45.99	22.97
Methylguaiacol	nd	nd	3.13	3.72	8.33	11.41	6	8.61
Syringaldehyde	12.76	17.04	42.76	82.18	170.4	276.6	67.24	111.47
Syringol	133.46	37.75	94.35	49.55	265.47	110.8	174.4	144.03
*trans-*Whiskylactone	3.01	2.92	23.01	57.99	7.81	14.98	4.04	4.64
Vanillin	19.4	19.4	34.13	64.14	92.09	108.9	36.31	74.9

**Table 5:** The influence of wood chips from different tree species on the concentration (μg/L) of compounds shaping the aroma of beers.

Sample	Control	Amburana	Balsam	Cariniana	Chestnut	Oak
5-Hydroxymethylfurfural	1540	3170	2080	850	3130	2840
Furfural	1820	1980	2930	1180	4280	110
Guaiacol	10	180	30	180	210	140
Syringaldehyde	930	2760	2230	3040	2760	2920
Vanillin	170	2330	4520	3820	2140	1020

**Table 6:** Concentrations of selected wood-derived compounds in whisky [176,177].

Compound	Concentration [μg/L]
4–Ethylguaiacol	0.74–380
4–Methylguaiacol	0.59–300
4–Vinylguaiacol	9.77–5000
5–Methylfurfural	6.88–3522
Acetovanilone	4.70–2405
*cis*–Whisky lactone	5.69–2916
Furfural	13.86–7098
Eugenol	0.98–500
Guaiacol	1.31–670
HMF	13.47–6895
Syringaldehyde	3.16–1620
*trans*–Whisky lactone	5.70–2916
Vanillin	13.93–7130

## Data Availability

No new data were created or analyzed in this study. Data sharing is not applicable to this article.
